# Rapid and Progressive Regional Brain Atrophy in CLN6 Batten Disease Affected Sheep Measured with Longitudinal Magnetic Resonance Imaging

**DOI:** 10.1371/journal.pone.0132331

**Published:** 2015-07-10

**Authors:** Stephen J. Sawiak, Sunthara Rajan Perumal, Skye R. Rudiger, Loren Matthews, Nadia L. Mitchell, Clive J. McLaughlan, C. Simon Bawden, David N. Palmer, Timothy Kuchel, A. Jennifer Morton

**Affiliations:** 1 Department of Physiology, Development and Neuroscience, University of Cambridge, Downing Street, Cambridge, UK; 2 Wolfson Brain Imaging Centre, University of Cambridge, Box 65 Addenbrooke’s Hospital, Hills Road, Cambridge, United Kingdom; 3 South Australian Research and Development Institute, Roseworthy, South Australia; 4 Preclinical, Imaging and Research Laboratories, South Australian Health and Medical Research Institute, Gilles Plains, South Australia; 5 Department of Molecular Biosciences, Faculty of Agriculture and Life Sciences and Batten Animal Research Network, Lincoln University, Lincoln, New Zealand; University of Sydney, AUSTRALIA

## Abstract

Variant late-infantile Batten disease is a neuronal ceroid lipofuscinosis caused by mutations in *CLN6*. It is a recessive genetic lysosomal storage disease characterised by progressive neurodegeneration. It starts insidiously and leads to blindness, epilepsy and dementia in affected children. Sheep that are homozygous for a natural mutation in *CLN6* have an ovine form of Batten disease Here, we used *in vivo* magnetic resonance imaging to track brain changes in 4 unaffected carriers and 6 affected Batten disease sheep. We scanned each sheep 4 times, between 17 and 22 months of age. Cortical atrophy in all sheep was pronounced at the baseline scan in all affected Batten disease sheep. Significant atrophy was also present in other brain regions (caudate, putamen and amygdala). Atrophy continued measurably in all of these regions during the study. Longitudinal MRI in sheep was sensitive enough to measure significant volume changes over the relatively short study period, even in the cortex, where nearly 40% of volume was already lost at the start of the study. Thus longitudinal MRI could be used to study the dynamics of progression of neurodegenerative changes in sheep models of Batten disease, as well as to assess therapeutic efficacy.

## Introduction

Batten disease is the term by which the neuronal ceroid lipoficinoses (NCL), a group of rare genetic lysosomal diseases, are commonly known [1]. They are the most prevalent group of children's progressive encephalopathies and account for 1/12,500 deaths in the USA [2]. NCLs are caused by many mutations in a diverse set of genes, with autosomal recessive mutations in *CLN5* and *CLN6* causing late-infantile variants [3,4]. There are two other genes in which mutations lead to so-called variant late infantile forms. These are *CLN7* and *CLN8*. The original infantile, late- infantile, juvenile adult descriptions were made on the basis of presenting symptoms but subsequent studies have shown that individual genetic forms can present many ways; for instance there are both congenital and adult-onset *CLN10* cases.

Onset of late-infantile Batten disease and variants typically occurs between 18 months and eight years of age, with symptoms of delayed and then regressive motor function, blindness, dysarthria, ataxia and dementia followed by premature death, usually during the second decade of life (for references, see [1]). The time from diagnosis to the premature death of an affected child is typically at least 5 years, and the disease imposes a heavy burden on affected families. Treatments such as anticonvulsants offer some palliative relief, but they do not influence the outcome. There are no effective cures for Batten disease at present, and little understanding of how the mutations cause the neurological decline.

Brain pathology in Batten disease patients is dominated by cortical atrophy, with the degree and progression of brain atrophy depending on the type and duration of the disease. Accumulation of autofluorescent storage bodies in most cells (for references see [1,5]). In the sheep under study here, as well as in many forms of the human disease, these storage bodies are comprised primarily of specifically stored subunit c of mitochondrial ATP synthase. However, storage body accumulation can occur independent of brain pathology (and also occurs throughout most visceral tissues, none of which show losses, of function). Thus we have a limited idea of how and when the mutant protein causes brain pathology and how this relates to symptomatic manifestation. It is difficult to conduct intensive studies on children with progressive brain diseases, particularly when infants. But without such studies, key questions such as (i) how/where the disease starts, (ii) how rapidly it progresses, (iii) whether progression is uniform across the brain and (iv) whether therapies can slow the progression of the disease once it has started, must remain unanswered. An understanding of the relationship between symptoms and brain pathology is critical if we are to gain an insight into how these diseases might be treated.

A number of natural animal models of Batten disease exist (for reviews see [6–9]). One of these is the South Hampshire sheep carrying a mutation in *CLN6* [10–12] that we used in this study. CLN6 sheep show the clinical hallmarks of an ovine version of late-infantile Batten disease, with characteristic storage body accumulation, neurodegeneration, abnormal behaviour and blindness [10,13–15]; for other references see [8]. They therefore represent an excellent model in which to measure progressive changes in brain structure. One obvious way of doing this would be to use magnetic resonance imaging (MRI), since it is non-invasive, and longitudinal MRI studies offer powerful means of tracking the progression of disease [16]. With subjects serving as their own controls, statistical power is increased considerably compared to that of cross-sectional studies. We have previously conducted *in vivo* MRI scans on a sheep model of Huntington disease [17]. To our knowledge however, MRI has not been used to track progressive degenerative changes in sheep with neurological disorders. Furthermore, there are no published MRI studies following any of the sheep models of Batten disease.

The aim of this study was to determine whether or not longitudinal MRI could be used to track *in vivo* the progressive brain atrophy in Batten disease affected sheep. We conducted *in vivo* MRI scans of the brains of 4 unaffected heterozygous carriers of *CLN6* mutation (here after described as control sheep) and 6 homozygous Batten disease affected sheep (hereafter described as Batten disease affected sheep) over a period of 5 months. We found that measurable brain atrophy in Batten disease affected sheep continued throughout the study period, not only in cerebral cortex (that already showed massive deterioration at the beginning of the study), but also in brain regions that were normal at the beginning of the study. Thus longitudinal MRI in sheep is both feasible and sensitive, and gives insight into the progression of the disease.

## Materials and Methods

### Study design

The objective of the research was to determine if (1) longitudinal scans could be conducted on animals with overt signs of neurodegenerative disease; (2) progressive atrophy was measurable in animals in which frank atrophy had already occurred; (3) progression of regional atrophy could be detected with longitudinal scanning. The affected animals showed signs of disease at the first time point, so the scans could not be conducted blind to genotype. Nevertheless, all MRI data was coded for analysis blind to genotype by another investigator (SJS) and the full details of each sheep were concealed until the data had been fully processed. Despite the evident cortical atrophy making groups obvious, analysis proceeded by automated scripts that handled each dataset identically with the same parameters so there was no possibility of introducing bias.

Sample size was 6 Batten disease affected sheep and 4 normal sheep (N = 10 in total). Such a study had not been conducted previously, so a power analysis was not possible. All data from all animals were included.

Rules for terminating the study of any individual sheep were defined by humane end points. One animal had seizures in the scanner during its first scan and was euthanized. After this, all procedures were reviewed, and the anaesthesia changed for all subsequent scans to an anaesthetic that would prevent further occurrences of scanner-induced seizures (probably due to noise). The endpoint was prospectively planned as the completion of 4 sequential scans.

### Animals

Animals were derived from 32 frozen embryos that were shipped from Lincoln University to South Australian Research and Development Institute where they were implanted into primed ewes. Of the resulting 12 pregnancies, 10 lambs survived. Animals of both sexes were used (N = 6 male; 4 control, 2 Batten disease affected), N = 4 female; all Batten disease affected). Males were castrated at 6 weeks. After weaning, all sheep were maintained in a single flock outdoors with *ad libitum* access to water and feed. Batten disease affected sheep were housed in covered pens for the last few months of the study. A routine clinical assessment was carried out daily on all Batten disease affected sheep to monitor health, as well as seizures.

All of the sheep were given MRI scans, with their first baseline scan conducted at ~16 months. Nine sheep survived for the duration of the experiment and were each scanned four times; one Batten disease affected sheep was euthanized shortly after its first scan (see below). Repeated scans were performed at baseline +7±2 weeks, baseline +12±1 weeks and baseline +18±2 weeks (mean ± standard deviation; S1 Table). All other sheep were killed by overdose of phenobarbitone at the end of the experiment. The animals were scanned at age 17.9±0.8, 19.6±0.5, 20.8±0.6, 22.1±0.6 months respectively and the standard deviation between scans for individuals was always less than 2 weeks. The interval between scans was taken into account in the analysis and an identical imaging protocol was used for each animal at each stage.

Images from ten sheep were used for voxel-based morphometry (VBM) analysis at baseline and to produce the study templates. For the longitudinal statistics, the euthanized sheep was excluded.

All studies were carried out in South Australia according to the Australian Code of Practice for the Care and Use of Animals for Scientific Purposes (2004) and under the approvals granted by the Primary Industries and Regions South Australia (PIRSA) (#18/10) and South Australia Pathology (#144/12) Animal Ethics Committees.

### Anaesthesia

For the conduct of MRI scans of sheep, anaesthesia is induced with diazepam 0.3mg/kg i.v. and ketamine 5mg/kg i.v., intubated and maintained with isoflurane at 2–2.5%, 5 l/min O_2,_ a ventilation tidal volume of 450–500l, and a respiratory rate of 12–15 breaths per minute. End tidal CO_2_ concentration is used to monitor ventilator adequacy. Because the third sheep scanned (No. 945) had a convulsive seizure in the scanner and then again in the recovery pen, and did not respond to intravenous doses of diazepam to control the seizures adequately, it was humanely killed on welfare grounds. For all subsequent scans sheep anaesthesia was induced with intravenous diazepam (0.3mg/kg), then 4% isoflurane in oxygen by face mask, and maintained with isoflurane at 2–2.5%, 5 l/min O_2_ via a semi-close circle absorber system driven by a pressure cycled ventilator (SV2500, Surgivet). Ketamine was eliminated from the protocol in order to remove its possible extrapyramidal effects.

### Image acquisition

Sheep were scanned supine in a 1.5T Siemens Sonata system using a variable flip angle 3D T2-weighted sequence (TR/TE 3500/355mm, echo train length 209, field of view 205mm, matrix 256×256×160 giving an isotropic resolution of 800μm with a single excitation). Signal transmission and excitation were performed with the manufacturer CP knee/extremity coil (Siemens Medical).

The scan sequences were designed to give sufficiently high-resolution data, in a reasonable time, with optimal contrast between grey and white matter and good signal-to-noise ratios.

### Voxel-based morphometry at baseline

Templates for VBM were created following the same procedure we used for the mouse brain [18]. Briefly, brain images were iteratively registered to a common template formed from the average of the warped images using SPM8 (Wellcome Trust Institute of Neurology, University College London, UK) with the SPMMouse toolbox (http://www.spmmouse.org). The unified segmentation algorithm of SPM8 was used [19] with DARTEL [20] to produce high-resolution transformations bringing the sheep into a common stereotactic space. The resulting warped and modulated grey and white matter images were smoothed with a 400μm isotropic Gaussian smoothing kernel and group wise differences at baseline were identified with a two-tailed t-test performed at each voxel. To determine statistical significance while controlling for type I errors likely due to multiple comparisons, an adjusted p-value was calculated using the family-wise error based on random field theory at p < 0.05.

### Longitudinal study

Each MRI was warped to create an average image at the median time point of scans for that animal, separately for each subject, using symmetric diffeomorphic modelling [21]. This framework is designed for group-wise intra-subject modelling calculating both rigid-body and non-linear registration with correction for intensity inhomogeneity. Processing was performed in SPM12b with default parameters including automatic estimation of the noise background, bias regularisation of 10^6^ and regularisation parameters of zero absolute displacement and membrane energy, 100 for bending energy and 25 for linear elasticity mu and 100 for the penalty on velocity divergence, lambda. The resulting template structural images at the average time point were segmented and registered together with DARTEL, following the same process as the baseline study. These transforms were applied to the divergence of velocity maps to assess the rate of change of volume on a voxel-wise basis in a common stereotactic space for comparison.

### Statistical Analysis

Maps of longitudinal rate changes were compared with voxel-wise Student's t-tests using SPM8. The false-discovery rate was used to control type I errors expected due to multiple comparisons with a threshold of pFDR<0.05 [22].

To clarify the longitudinal changes in plotting volumes for each sheep, volumes were normalised by adding a constant offset to the volume of each structure at each time point, for each sheep, so that baseline volumes were the same for all of the sheep within each group. The solid lines on each graph show a linear fit to these points and the variability of the data is shown by shading a region equal to two standard deviations of the post-baseline data above and below the line.

Statistical significance for longitudinal changes in the regions of interest shown was determined by comparing the product-moment coefficient of the adjusted data after a Fisher's z-transformation with p < 0.05 used as a threshold for rejecting the null hypothesis, without controlling for multiple comparisons.

## Results

### Batten sheep clinical phenotype

**Fig 1:**
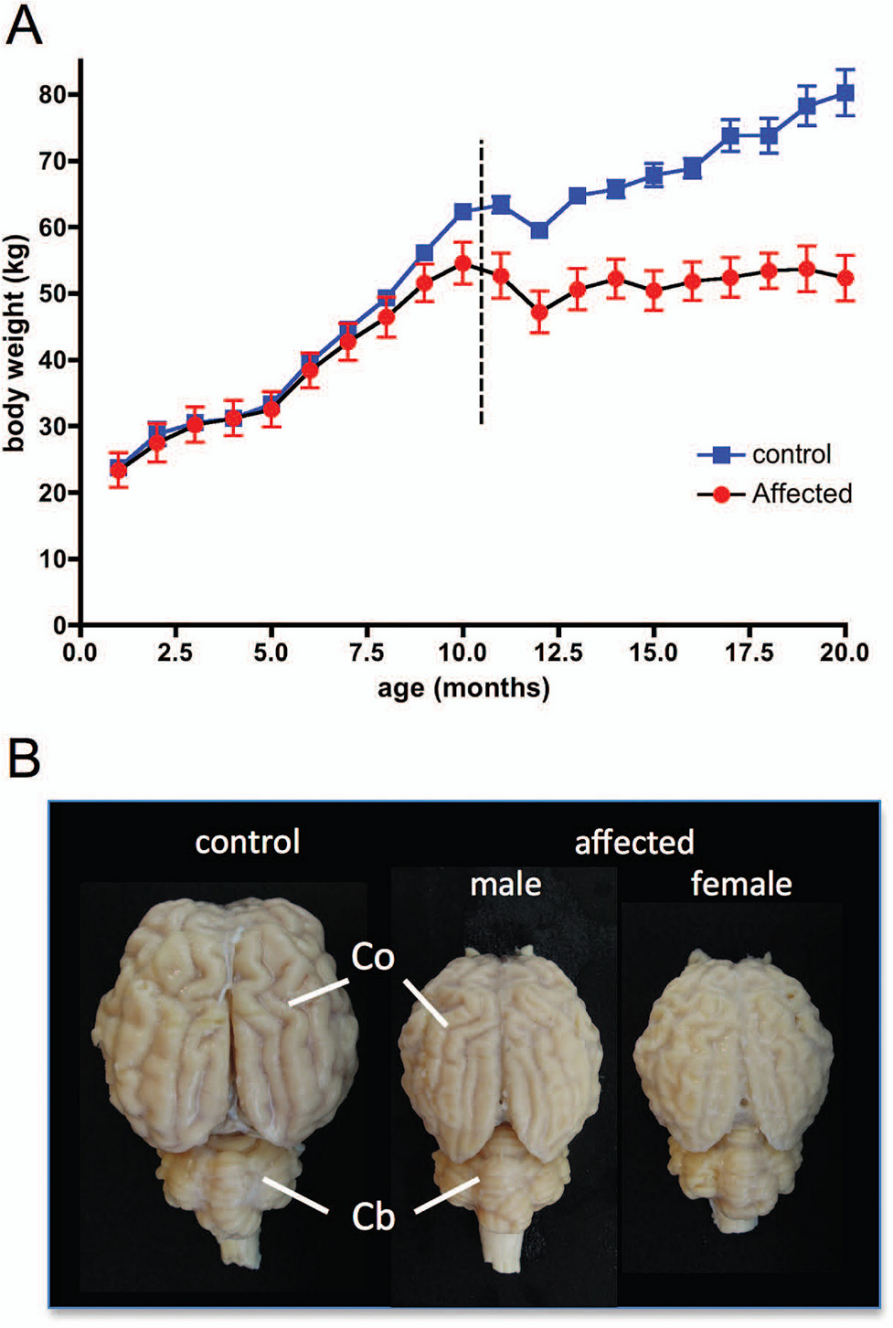
Batten disease affected sheep body weight changes and *post mortem* brain appearance. (A) Mean (± SEM) body weight of control (blue) and Batten disease affected sheep (red) from birth to 20 months of age shows that growth of Batten disease affected animals stopped at around 14 months. The dotted line indicates the time of transfer from the home farm to the MRI facility. (B) Comparison of gross anatomy of brains from a control sheep (left) and the least affected wether (male, 939), and least affected ewe (female, 937) (centre and right respectively) shows pronounced atrophy of the cerebral cortex (Co) with relative sparing of the cerebellum (Cb). Brains were fixed with 4% paraformaldehyde. Size bar in B represents 3cm.

Body weights of the Batten disease affected sheep were the same as those of control sheep until they were 10 months old, after which their rate of growth slowed relative to that of unaffected sheep (Fig 1A). All of the sheep lost weight when they moved to the MRI facility (Fig 1A, dashed line) following a change in diet and the onset of high summer, when grazing is limited. (The sheep were housed outdoors until after they had their first scan.) The control sheep recovered their body weight within a few weeks, but growth of the Batten disease affected sheep stopped. By the time the sheep were moved to the MRI facility, all Batten disease affected sheep showed clear signs of disease.

Once the sheep were moved to the MRI facility, records of abnormal behaviour were kept from daily observations. One affected sheep showed manifest seizures before transfer to the MRI facility and another one other sheep had 2 episodes of manifest convulsive seizures. All of the remaining affected sheep showed the abnormal motor behaviour characteristic of this line of sheep [11], but not convulsive seizures. The least affected sheep (939) was behaviourally indistinguishable from the control sheep at baseline (the first scan), and showed minimal clinical signs at the final scan. Nevertheless, massive neocortical atrophy was evident in this sheep at *post mortem* compared to the control sheep (Fig 1B). *Post mortem* examination of the brains of all affected sheep confirmed MRI findings, showing significant atrophy of the cerebral cortex with sparing of the cerebellum. The brains shown in Fig 1 are those of a control sheep (936), the least clinically-affected wether (male, 939), and least clinically-affected ewe (female, 937).

### Longitudinal MRI scanning in sheep

**Fig 2:**
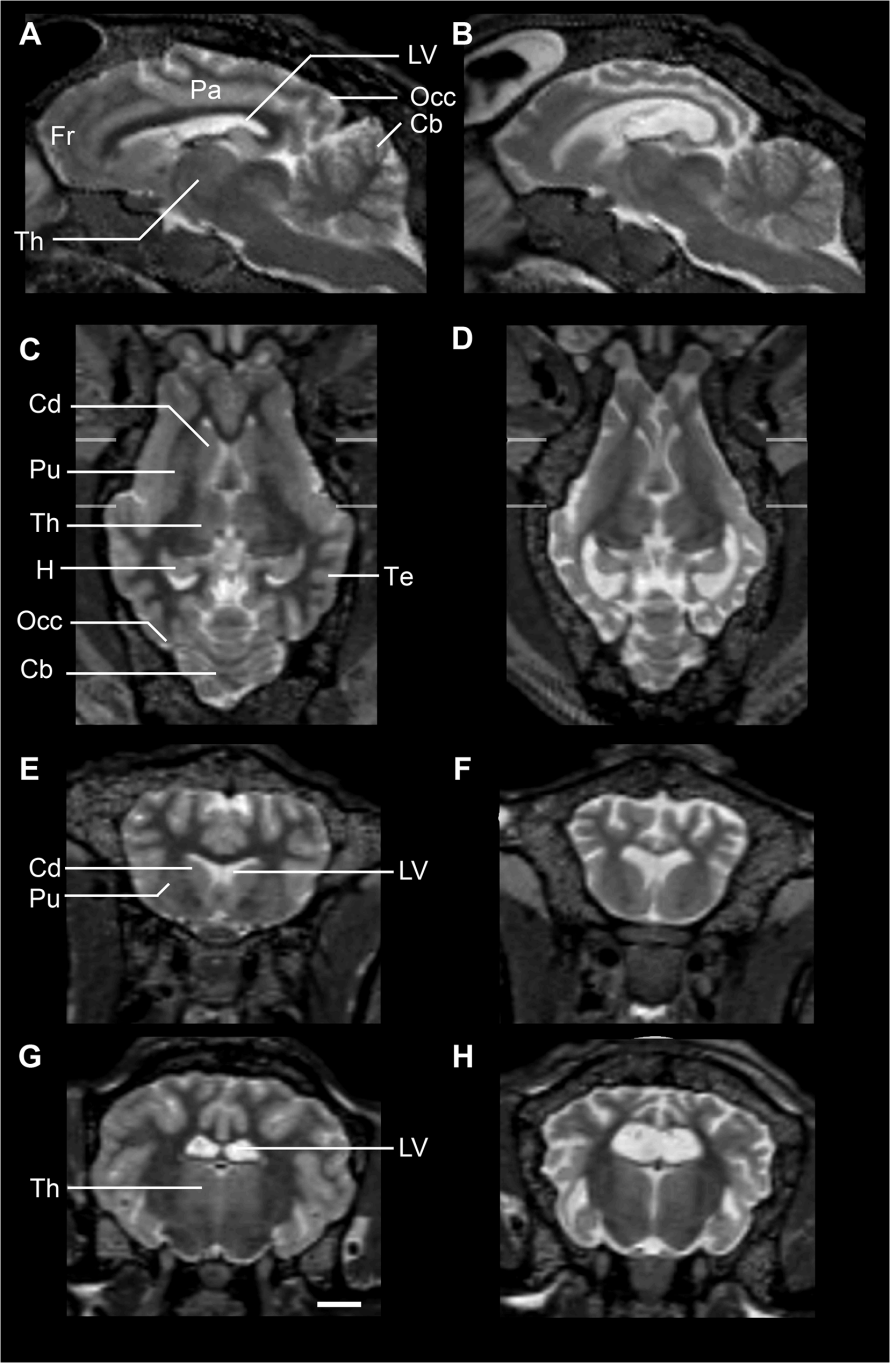
MRI of Batten disease and control sheep. MRI sections (T2) at baseline of a typical unaffected control (A, C, E, G) and a Batten disease affected sheep (B, D, F, H) in sagittal (A-B), horizontal (C-D) and coronal views at two different levels (E-H). Significant brain atrophy is evident in the affected sheep, particularly in the cortex, with concomitant enlargement of the ventricles. Scale bar is 1cm. Abbreviations: Fr frontal cortex, Th thalamus, Pa parietal cortex, LV lateral ventricles, Occ occipital cortex, Cb cerebellum, Cd caudate, Pu putamen, H hippocampus, Te temporal lobe.

Baseline scans were done when the animals were 16.5–18 months old (S1 Table), by which age they all had clinical signs, although these varied markedly in severity (S2 Table). Irrespective of severity of symptoms, MRI of all Batten disease affected sheep brains showed pronounced cortical atrophy compared to controls at baseline. Typical examples of scans are shown in Fig 2. The difference in brain volume is obvious between the control and affected animals, particularly in the cortex. By contrast, the cerebellum appears to be similarly sized in both animals, with well-preserved morphology. This is consistent with previous findings where the cerebellum is preserved in sheep models of Batten disease (8). Subcortical structures such as the caudate nuclei and putamen also look relatively normal, although these regions are smaller in the Batten disease affected sheep, and there is an accompanying increase in the size of the lateral ventricles.

### Progressive regional atrophy of brain in Batten disease affected sheep

**Fig 3:**
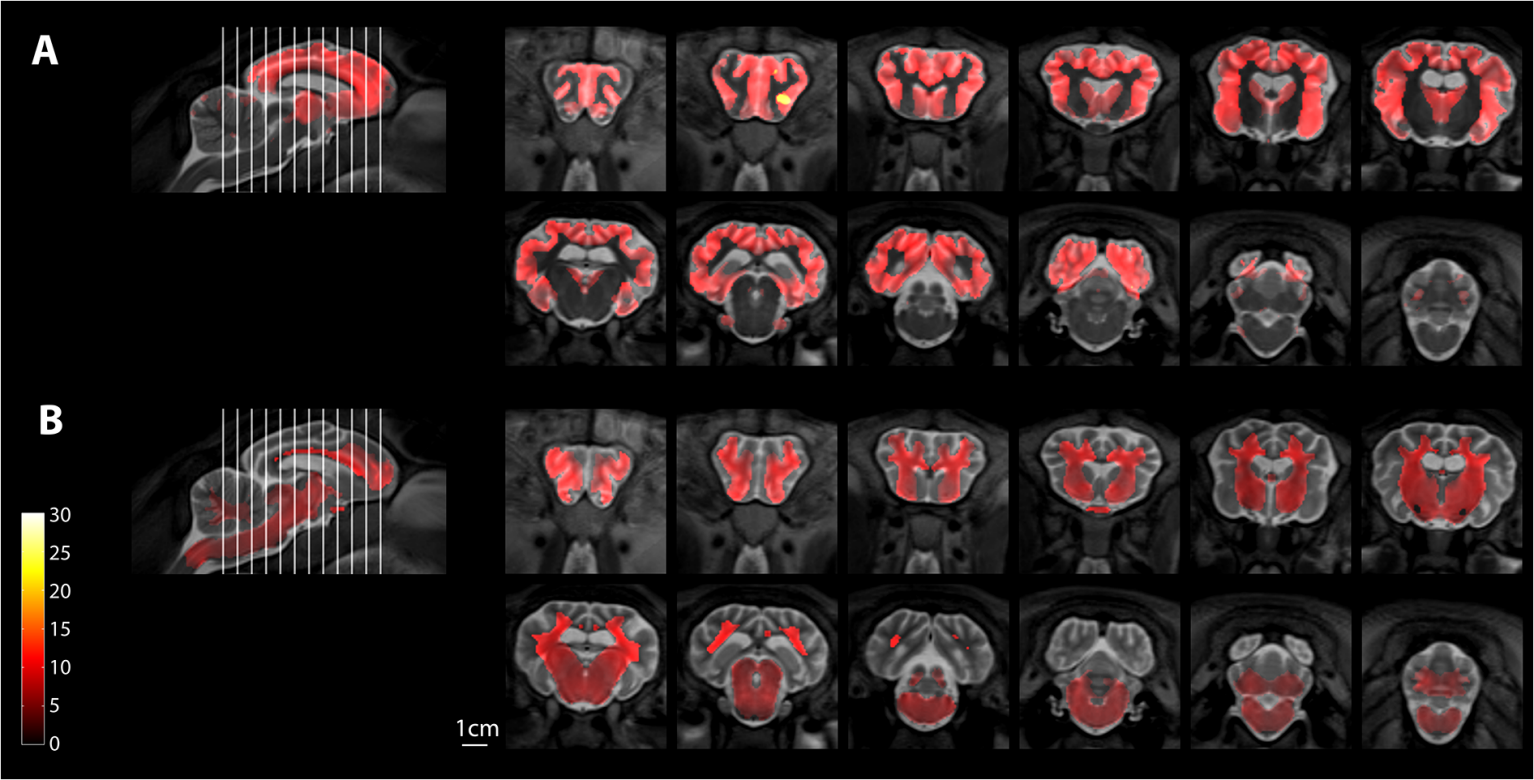
Voxel-based morphometry results showing differences between control sheep and Batten affected sheep, at the baseline scan. Coronal sections are pseudocoloured to show changes in grey matter (A), or white matter (B). The colour bar shows Student’s t-score with 8 degrees of freedom. The position of the sequential coronal sections indicated by the lines on the accompanying sagittal section.

**Fig 4:**
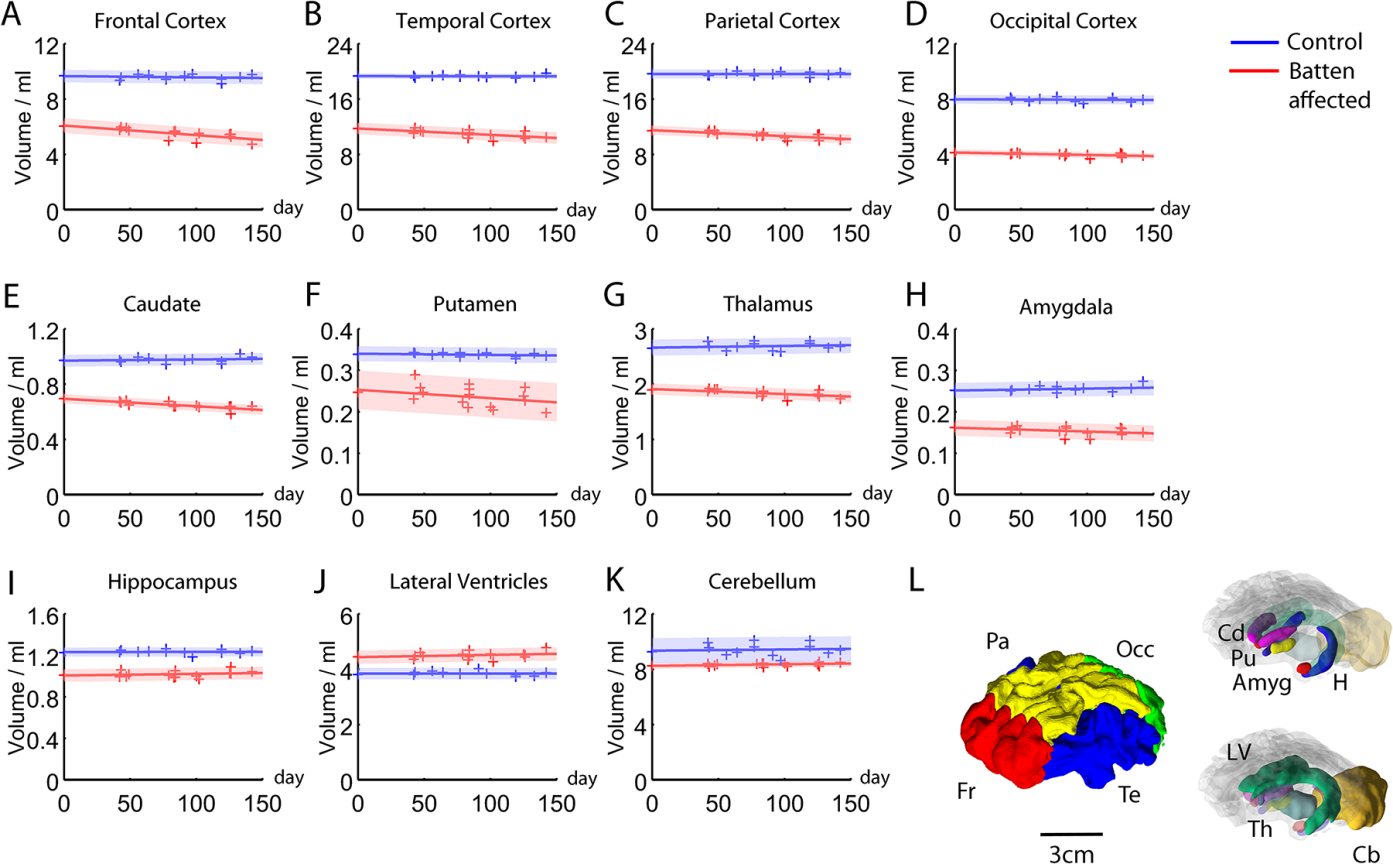
Regional volume changes in Batten disease sheep. Volumes are shown for control (blue) and Batten affected sheep (red) of cortex (A-D), basal ganglia (E-F), thalamus **(G),** amygdala (H), hippocampus (I), lateral ventricles (J) and cerebellum (K) with 3D reconstructions of the regions measured shown in L. Day 0 is the day of the baseline scan. Solid lines show a least-squares linear fit, the shaded region extends two standard deviations either side of the line. Volumes were normalised as described in the text.

Voxel-based morphometric analysis of the whole brain from the group at baseline is shown in Fig 3. Significant differences in both grey (upper panels) and white matter (lower panels) between the control and affected sheep were seen in most brain structures, the notable exception being the cerebellum. There were also significant differences in regional volumes in most areas measured at baseline (Fig 4). The notable exception was the cerebellum, where there was a trend, but no statistical difference in the mean volume at baseline between Batten disease affected sheep (8.3±0.6ml) and control sheep (9.4±0.2ml; p = 0.12).

Post mortem atrophy is pronounced in CLN6 affected sheep, and we can detect changes through CT scanning around 4 months of age for some individuals and in all by 8–9 months (unpublished data, NM and DP). We wondered if, despite the fact that there was already significant structural change in the Batten disease affected sheep at baseline, we would be able to detect further degeneration after subsequent scans. S1 Fig. shows representative slices through the brains of three sheep (one unaffected, and the least- and worst-affected Batten disease affected sheep) at each of the four different scan times. Even though there were only five months from the first scan to the last, progressive atrophy can be seen clearly in the affected animals, particularly in the cortex and caudate.

**Fig 5:**
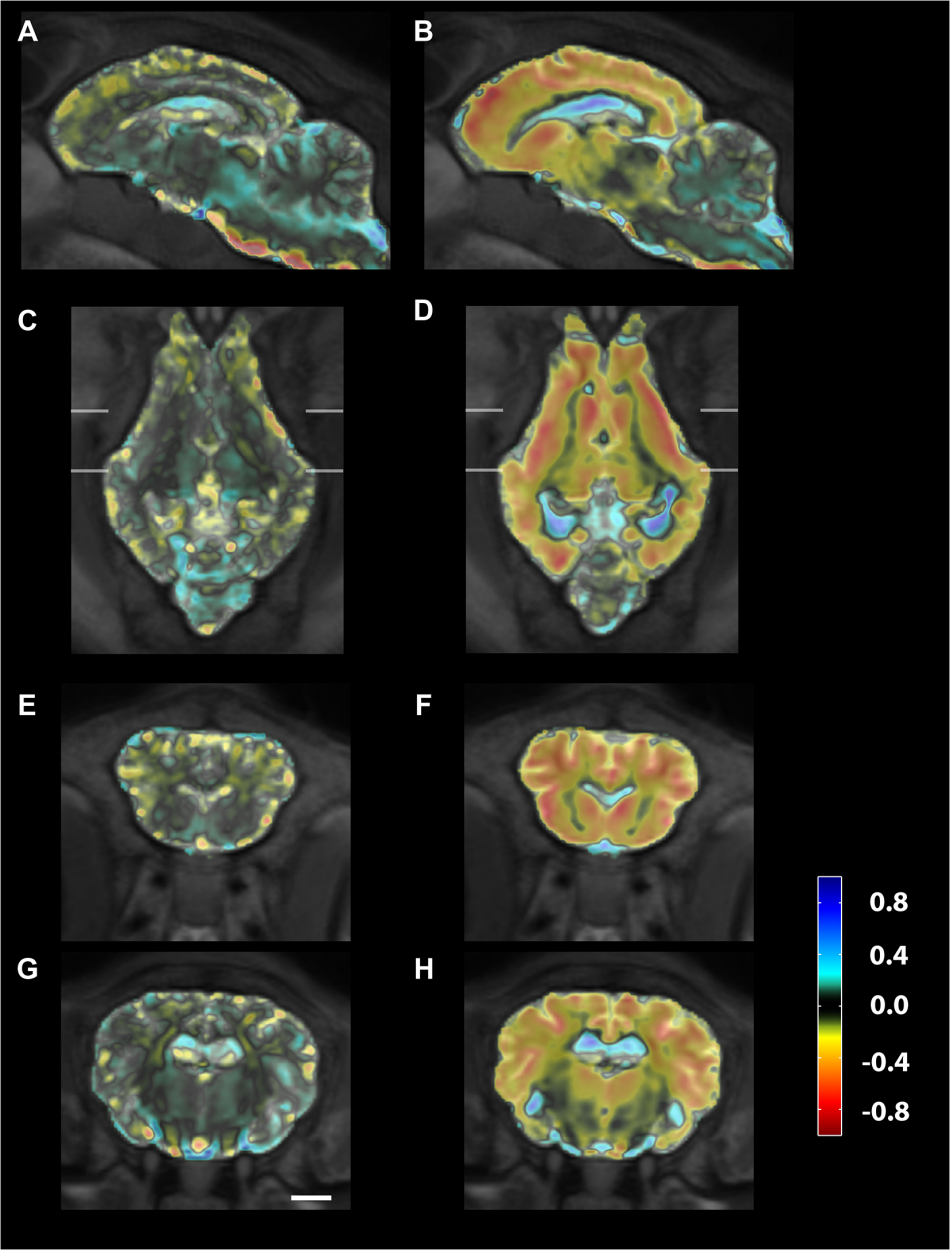
Map of mean volume changes in Batten disease affected sheep. Volume changes are shown for control sheep (A, C, E, G) and Batten disease affected sheep (B, D, F, H) in sagittal (A, B)), horizontal (C, D) and two coronal planes (E-H), one at the level of the striatum (E, F), the other at the level of the thalamus (G,H). Scale bar in G is 1cm and the colour scale indicates volume change as ml/year.

Longitudinal changes were assessed quantitatively by comparing the divergence of velocity fields for the registration parameters derived in the image registration process. Mean maps of local volume changes per year are shown for each group of sheep in Fig 5. In Batten disease affected sheep, it is clear that progressive atrophy is occurring throughout the cortex and subcortical structures with corresponding enlargement of the fluid spaces. Changes visible in the control sheep are mainly small fluctuations around fluid spaces. On average over the cortex, controls showed small but detectable grey matter volume reductions of (2±4)% per year whereas cortical atrophy in Batten disease affected animals was (31±10)% per year (mean ± SD).

We checked to see if there was a correlation between brain atrophy and clinical stage, as assessed by age of onset, body weight/ seizures. There was a tendency for a negative correlation between seizures and cortical volume (Pearson’s *r* = -0.6), but this was not statistically significant (p = 0.3).

### Atrophy starts in cortex but progresses to subcortical structures

Significant progressive atrophy in the frontal, temporal and parietal cortices was observed during the study, even though 40% of the cortical volume had been lost by the time the study started (Fig 4, S3 Table). There was a measurable, but less pronounced atrophy in caudate, putamen and amygdala at baseline, and atrophy in the thalamus and caudate progressed during the study period, with the greater rate of atrophy found in the caudate. There was a trend to enlargement of the lateral ventricles during the study, but this was not significant due to high individual variability. The only parts of the brain that we measured that did not show atrophy were the hippocampus and the cerebellum, which was remarkably spared (Figs 1B, 4 and 5). Some areas of the white matter within the cerebellum showed a detectable difference with VBM (Fig 3) but these differences are subtle compared with the gross effects seen elsewhere. No laterality was observed. Effects were similar in both hemispheres, both at baseline and during the progression of disease.

**Fig 6:**
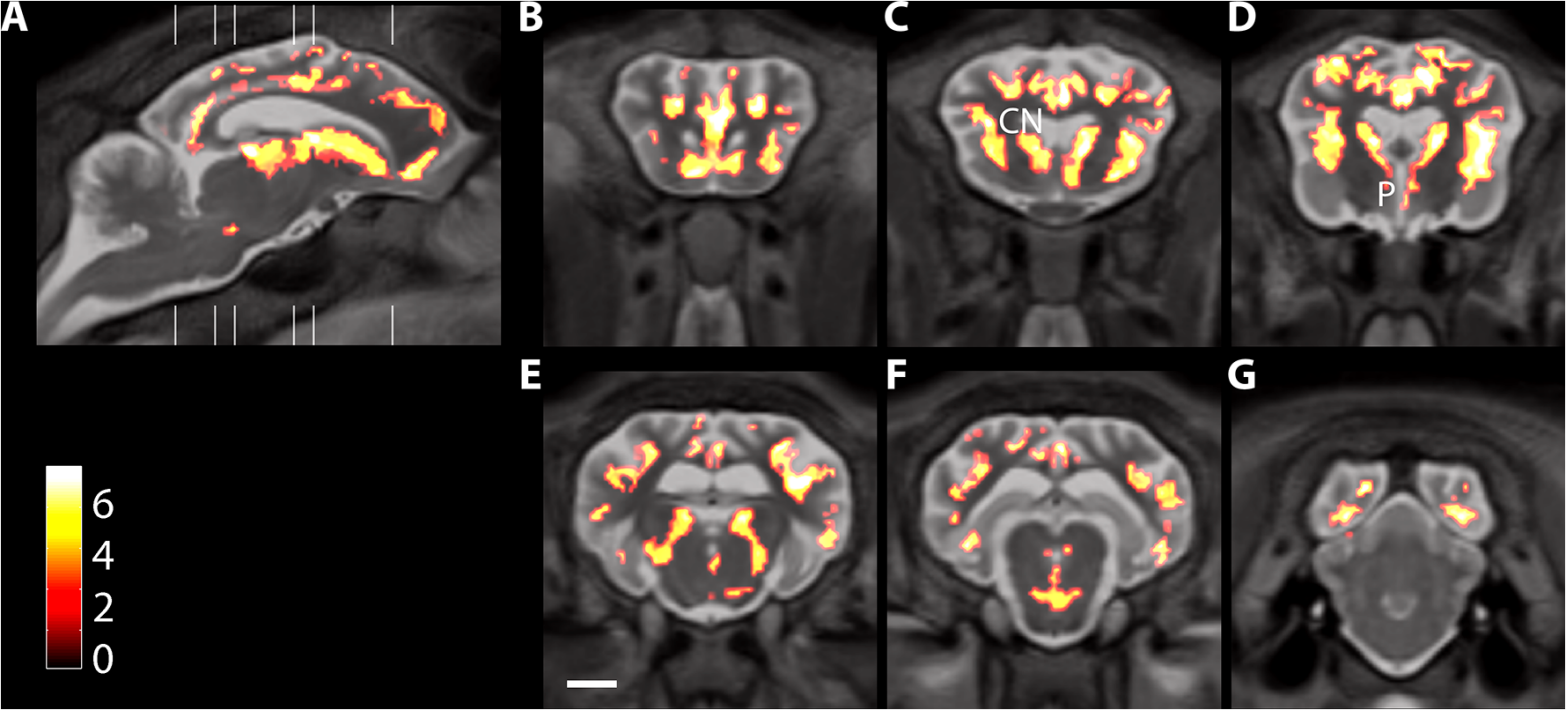
Representative sections showing significant differences in longitudinal rates of change between control and Batten disease affected sheep. Significant changes in Batten disease sheep are shown on representative pseudocoloured sagittal and coronal sections. White lines on the sagittal section indicate the position of the coronal sections on the right. Colour bar indicates Student’s t-score with seven degrees of freedom. All changes shown are significant at p < 0.05 adjusted for multiple comparisons by controlling the false-discovery rate. Scale bar in E is 1 cm. (The full data set is shown in S2 Fig.)

From the maps of significant changes that occurred during the study (Fig 6), region-specific atrophy is easily identified in anatomically well-defined regions such as the caudate and putamen. These maps also show changes that suggest there are small nuclei that degenerate in Batten disease. For example, in the brainstem image in Fig 6E, there are significant changes in volume in an area of the midbrain that aligns with the substantia nigra (Fig 6E, S2 Fig).

## Discussion

We conducted a longitudinal MRI study of brain structure and volume in a small group of CLN6 Batten disease affected sheep. We found significant atrophy present in all sheep at the baseline scan, regardless of clinical presentation. This is consistent with the finding from *post mortem* analyses showing that cortical atrophy in CLN6 sheep begins many months before clinical signs are present [10,11,14,15]. We conducted four scans over a period of four to five months, and found that atrophy continued measurably in this time in multiple regions, including the cortex, the region that was most affected at baseline. This study shows that 1) multiple MRI scans can be conducted in sheep affected by a severe and progressive neurological disease; 2) only small numbers of animals are needed for statistical significance; 3) in Batten disease sheep atrophy continues measurably in degenerating regions of the brain, even over the relatively short period of our study; 4) there is a differential time course of atrophy in different parts of the brain, with both magnitude and rate of atrophy varying between regions.

Changes in MRI in affected Batten disease sheep brains resembled MRI findings in late-infantile Batten disease patients, all of whom show typically diffuse mild to moderate cerebral atrophy [23–27]. There have only been a few case reports on late-infantile Batten disease caused by mutations in *CLN6* that include MRI [28], and in these the pathology varied markedly between patients, even in consanguineous patients carrying the same gene mutation. Interestingly, in one of these patients described by Guereirro et al., the periventricular white matter and posterior limb of internal capsules along with deep cerebellar white matter showed ‘high T2 signal with hazy appearances’. Subcortical hyperintensity was also reported in 75% of late-infantile NCL in a retrospective study (see Table 2 in [26]), although these patients were classified according to age-at-onset, rather than by genotype. Hyperintensity of white matter was seen in the CLN6 Batten disease affected sheep, although this is not as pronounced in the periventricular region in sheep as it is described in patients. It would be interesting to see this was a feature that also differed in the ovine models of Batten disease caused by different mutations.

Our study highlights both the usefulness and sensitivity of longitudinal MRI studies, and the feasibility of using MRI to study progressive pathology in sheep with neurological diseases. Longitudinal MRI gave insight into pathology that might have been missed in a cross-sectional study. For example, it was interesting to note that after the cortex, the most affected areas were basal ganglia nuclei (caudate and putamen, and then the amygdala), where no neuronal loss has been seen in a 2 year old CLN6 sheep [14]. Subcortical pathology has not been well mapped in CLN6 Batten disease sheep, and basal ganglia pathology has not been shown in late-infantile disease caused by *CLN6* mutations [29], although it is pronounced in some other late-infantile forms [30]. As well as progressive atrophy, we noted that atrophy first became measurable in the thalamus during the period of study and hippocampal volume showed a trend towards a significant volume change, although this did not survive correction for multiple comparisons. These findings contrast with some of the literature on CLN6 sheep and patients with CLN6 mutations causing Batten disease. For example, neuronal loss in basal ganglia was not seen in a 2 year old CLN6 sheep [14] and basal ganglia pathology has not been shown in variant late-infantile disease caused by CLN6 mutations [29] although it is pronounced in some other late-infantile forms [30]. The thalamic changes we describe contrast with previous studies that report either no, or limited, histological changes in the thalamus in *CLN6* Borderdale Batten disease sheep from the same flock reared under different conditions [15], although thalamic atrophy is present in another *CLN6* (Merino) Batten disease sheep [12], the *nclf* mouse that has a natural disease-causing mutation in *CLN6* [31] and in some late infantile Batten disease patients. The reason for the discrepancy between our study and the previous studies on the Borderdale Batten disease affected sheep is not clear; it may simply be the case that our longitudinal study has greater sensitivity than case studies or cross-sectional studies, since the effect of inter-subject variability (that is a problem in studies using few subjects) is removed. Our study focussed on a single age point for the baseline scan and subsequent follow-up scans in a single genetic mutation (CLN6). The clear results of this study highlight the effectiveness of MRI for morphometric assessment in ovine NCL. It would be interesting to explore morphological changes in other ovine variants of NCL and also to examine a much wider range of ages, in a larger study.

Detailed statistical analysis of longitudinal MRI gave additional insights, such as the selective atrophy of deep layers of the cortex (Fig 6) as well as changes in the midbrain in region that map to the substantia nigra. This would be interesting to investigate in more detail histologically. While neurodegeneration in substantia nigra has not been described in CLN6 disease (either humans or sheep), changes in dopamine have been found in Batten disease caused by mutations in the *CLN2* gene in humans [32] and in a mouse model with a mutation in *Cln3* [33] as well as in other neuronal ceroid lipofuscinoses [34,35]. The relationship between disease pathology and clinical signs in the sheep is not established. Caudate atrophy would be expected to exacerbate loss of motor control, as well as to complicate cognitive decline. Thalamic degeneration might contribute to seizure generation and could also cause sleep abnormalities that are common at late stages in Batten disease [36]. We do not know if sleep abnormalities are present in CLN6 Batten disease sheep, although from the regions that are atrophied we predict that they would be present. Only the cerebellum was spared by the end of the study period, which is consistent with preserved balance and gait in the CLN6 Batten disease affected sheep.

In the present study that the sex ratios were not the same between control and Batten affected groups. Sexual dimorphism can be seen by MRI, but is a much smaller effect than the large scale differences we report here. Total brain volumes were ~30% lower in the male affected sheep compared to the male control sheep. Within the group of affected sheep, the four females had brains that were only 10% smaller than the male. Thus the effect of disease has a much stronger effect on brain size than the natural differences of sexual dimorphism. For the longitudinal study, each animal served as its own control, so sex was not a factor. An alternative to the analysis we have performed would be to normalise by overall brain volume before comparing the groups. Since so much of the brain is affected, however, this normalisation will tend to remove the appearance of disease effects and introduce artefactual findings in comparatively spared structures, especially the cerebellum.

The naturally occurring Batten diseases that exist in large animals make sheep ideal models in which to develop therapies such as gene therapy [37–40]. The sheep model would also be useful for studying pathology in more detail, for example the role of neurodegeneration in different regions during the course of disease. Now that we have established that longitudinal MRI can be conducted successfully in sheep, it should also be possible to do functional MRI studies. Using sheep could also help to answer important questions such as whether or not treating mid-late stage disease can prevent further deterioration that compromises quality of life. The progressive degeneration we see in the sheep suggests that any treatment that slows or halts the disease may be beneficial. For example, although nearly 40% of the cortex had atrophied by the time of our first scans, atrophy continued measurably in the cortex over the next 5 months, particularly in the deep layers. Furthermore, although the thalamus and hippocampus were not different from controls at base line, they started to show measurable atrophy during the study period. Preventing degeneration of subcortical regions may prevent or delay symptoms that are caused or exacerbated by degeneration in those areas. We suggest that MRI will be particularly useful for providing a ‘readout’ for determining the efficacy of treatments in sheep models, not only for CLN6 Batten disease, but also for other neurodegenerative diseases where sheep models exist. In addition to the Batten disease models discussed above, these would include natural sheep models of Gaucher disease [41], Hereditary lissencephaly and cerebellar hypoplasia [42], adult-onset Alexander disease [43] and Tay-Sachs disease [44] as well as the recently developed transgenic ovine model of Huntington disease [17,45]. Longitudinal MRI provides precise mapping of changes in individual animals, and considerably increases the power of such studies while requiring fewer individuals than would be needed in a cross-sectional study. With the sensitivity to detect reliably changes over a short period, MRI combined with sheep models of human disease will provide an important platform for the investigation of potential therapies and interventions that have a realistic prospect for translation to patients.

## Supporting Information

S1 Fig Progressive atrophy of brain in Batten disease. MRI sections showing longitudinal progression in a control sheep (A) and the least- (B) and worst-affected (C) Batten disease affected sheep. To aid comparison in the figure, gridlines were drawn to frame the grey matter surface of each of brain at the last time point, and these lines are reproduced on the earlier scans for comparison. Gridlines on each set for scans are a box enclosing the grey matter surface on the final scan reproduced at each of the earlier time points. Scale bar is 1cm.
(TIF)

S2 Fig Significant differences in longitudinal rates of change between control and Batten affected sheep (full data set). Colour bar indicates Student’s t-score with seven degrees of freedom. All changes shown are significant at p < 0.05 adjusted for multiple comparisons by controlling the false-discovery rate.
(TIF)

S1 Table Timeline of MRI scans. (DOCX)

S2 Table Seizure onset and severity in homozygous affected Batten sheep. (DOCX)

S3 Table Regional volume changes in Batten disease sheep. (DOCX)

## References

[1] Mole SE, Williams RE, Goebel HH. The neuronal ceroid lipofuscinoses (Batten disease) 2nd ed. ed. Oxford: Oxford University Press; 2011.

[2] Rider JA, Rider DL. Batten disease, a twenty-eight-year struggle: past, present and future. Neuropediatrics. 1997;28(1):4–5. 10.1055/s-2007-973653 . 9151308

[3] Kousi M, Lehesjoki AE, Mole SE. Update of the mutation spectrum and clinical correlations of over 360 mutations in eight genes that underlie the neuronal ceroid lipofuscinoses. Human mutation. 2012;33(1):42–63. 10.1002/humu.21624 . 21990111

[4] Mole SE, Williams RE, Goebel HH. Correlations between genotype, ultrastructural morphology and clinical phenotype in the neuronal ceroid lipofuscinoses. Neurogenetics. 2005;6(3):107–26. 10.1007/s10048-005-0218-3 . 15965709

[5] Special issue on The Neuronal Ceroid Lipofuscinoses or Batten Disease. Biochim Biophys Acta 2013;1832:1793–912. 10.1016/j.bbadis.2013.05.025 23727410

[6] Bond M, Holthaus SMK, Tammen I, Tear G, Russell C. Use of model organisms for the study of neuronal ceroid lipofuscinosis. Bba-Mol Basis Dis. 2013;1832(11):1842–65. 10.1016/j.bbadis.2013.01.009 . 23338040

[7] Palmer DN, Barry LA, Tyynela J, Cooper JD. NCL disease mechanisms. Bba-Mol Basis Dis. 2013;1832(11):1882–93. 10.1016/j.bbadis.2013.05.014 . 23707513

[8] Palmer DN, Tammen I, Drögemüller C, Johnson GS, Katz ML, Lingass F. Large animal models In: Mole SE, Williams RE, Goebel HH, editors. The Neuronal Ceroid Lipofuscinoses (Batten Disease). 2nd ed. London: Oxford University Press; 2011 p. 284–320.

[9] Weber K, Pearce DA. Large Animal Models for Batten Disease: A Review. J Child Neurol. 2013;28(9):1123–7. 10.1177/0883073813493666 . 24014507 PMC4009683

[10] Jolly RD, Janmaat A, West DM, Morrison I. Ovine ceroid-lipofuscinosis: a model of Batten's disease. Neuropathology and applied neurobiology. 1980;6(3):195–209. . 6772982 10.1111/j.1365-2990.1980.tb00290.x

[11] Jolly RD, West DM. Blindness in South Hampshire sheep: a neuronal ceroidlipofuscinosis. New Zealand veterinary journal. 1976;24(6):123 10.1080/00480169.1976.34298 . 1067509

[12] Tammen I, Houweling PJ, Frugier T, Mitchell NL, Kay GW, Cavanagh JA, et al A missense mutation (c.184C>T) in ovine CLN6 causes neuronal ceroid lipofuscinosis in Merino sheep whereas affected South Hampshire sheep have reduced levels of CLN6 mRNA. Biochimica et biophysica acta. 2006;1762(10):898–905. 10.1016/j.bbadis.2006.09.004 . 17046213

[13] Broom MF, Zhou C, Broom JE, Barwell KJ, Jolly RD, Hill DF. Ovine neuronal ceroid lipofuscinosis: a large animal model syntenic with the human neuronal ceroid lipofuscinosis variant CLN6. Journal of medical genetics. 1998;35(9):717–21. 9733028 10.1136/jmg.35.9.717 PMC1051422

[14] Oswald MJ, Kay GW, Palmer DN. Changes in GABAergic neuron distribution in situ and in neuron cultures in ovine (OCL6) Batten disease. European journal of paediatric neurology: EJPN: official journal of the European Paediatric Neurology Society. 2001;5 Suppl A:135–42. . 11588985 10.1053/ejpn.2000.0450

[15] Oswald MJ, Palmer DN, Kay GW, Shemilt SJ, Rezaie P, Cooper JD. Glial activation spreads from specific cerebral foci and precedes neurodegeneration in presymptomatic ovine neuronal ceroid lipofuscinosis (CLN6). Neurobiology of disease. 2005;20(1):49–63. 10.1016/j.nbd.2005.01.025 . 16137566

[16] Mills KL, Tamnes CK. Methods and considerations for longitudinal structural brain imaging analysis across development. Developmental cognitive neuroscience. 2014;9:172–90. 10.1016/j.dcn.2014.04.004 . 24879112 PMC6989768

[17] Morton AJ, Rudiger SR, Wood NI, Sawiak SJ, Brown GC, McLaughlan CJ, et al Early and progressive circadian abnormalities in Huntington's disease sheep are unmasked by social environment. Hum Mol Genet. 2014;23(13):3375–83. 10.1093/hmg/ddu047 . 24488771

[18] Sawiak SJ, Wood NI, Williams GB, Morton AJ, Carpenter TA. Voxel-based morphometry with templates and validation in a mouse model of Huntington's disease. Magnetic resonance imaging. 2013;31(9):1522–31. 10.1016/j.mri.2013.06.001 23835187 PMC3919157

[19] Ashburner J, Friston KJ. Unified segmentation. NeuroImage. 2005;26(3):839–51. 10.1016/j.neuroimage.2005.02.018 . 15955494

[20] Ashburner J. A fast diffeomorphic image registration algorithm. Neuroimage. 2007;38(1):95–113. Epub 2007/09/01. doi: S1053-8119(07)00584-8 [pii] 10.1016/j.neuroimage.2007.07.007 . 17761438

[21] Ashburner J, Ridgway GR. Symmetric diffeomorphic modeling of longitudinal structural MRI. Frontiers in neuroscience. 2012;6:197 10.3389/fnins.2012.00197 23386806 PMC3564017

[22] Genovese CR, Lazar NA, Nichols T. Thresholding of statistical maps in functional neuroimaging using the false discovery rate. Neuroimage. 2002;15(4):870–8. . 11906227 10.1006/nimg.2001.1037

[23] Alroy J, Braulke T, Cismondi IA, Copper D, Creegan M, Elleder C, et al CLN6 In: Mole SE, Williams RE, Goebel HH, editors. The Neuronal Ceroid Lipofuscinoses (Batten Disease). 2nd ed. London: Oxford University Press; 2011 p. 284–320.

[24] Autti T, Raininko R, Santavuori P, Vanhanen SL, Poutanen VP, Haltia M. MRI of neuronal ceroid lipofuscinosis. II. Postmortem MRI and histopathological study of the brain in 16 cases of neuronal ceroid lipofuscinosis of juvenile or late infantile type. Neuroradiology. 1997;39(5):371–7. . 9189886 10.1007/s002340050427

[25] Autti T, Raininko R, Vanhanen SL, Santavuori P. MRI of neuronal ceroid lipofuscinosis. I. Cranial MRI of 30 patients with juvenile neuronal ceroid lipofuscinosis. Neuroradiology. 1996;38(5):476–82. . 8837098 10.1007/BF00607283

[26] Jadav RH, Sinha S, Yasha TC, Aravinda H, Gayathri N, Rao S, et al Clinical, electrophysiological, imaging, and ultrastructural description in 68 patients with neuronal ceroid lipofuscinoses and its subtypes. Pediatric neurology. 2014;50(1):85–95. 10.1016/j.pediatrneurol.2013.08.008 . 24120650

[27] Vanhanen SL, Raininko R, Santavuori P. Early differential diagnosis of infantile neuronal ceroid lipofuscinosis, Rett syndrome, and Krabbe disease by CT and MR. AJNR American journal of neuroradiology. 1994;15(8):1443–53. . 7985561 PMC8334405

[28] Guerreiro R, Bras JT, Vieira M, Warrier V, Agrawal S, Stewart H, et al CLN6 disease caused by the same mutation originating in Pakistan has varying pathology. European journal of paediatric neurology: EJPN: official journal of the European Paediatric Neurology Society. 2013;17(6):657–60. 10.1016/j.ejpn.2013.04.011 23735787 PMC3847240

[29] Davidson BL, Cabrera-Salazar MA, Pearce DA. The neuronal ceroid lipofscuinoses: clinical features and molecular basis of disease In: Barranger JA, Cabrera-Salazar MA, editors. Lysosomal storage disorders. New York: Springer; 2007 p. 371–88.

[30] Simonati A, Santorum E, Tessa A, Polo A, Simonetti F, Bernardina BD, et al A CLN2 gene nonsense mutation is associated with severe caudate atrophy and dystonia in LINCL. Neuropediatrics. 2000;31(4):199–201. 10.1055/s-2000-7453 . 11071145

[31] Kielar C, Wishart TM, Palmer A, Dihanich S, Wong AM, Macauley SL, et al Molecular correlates of axonal and synaptic pathology in mouse models of Batten disease. Human molecular genetics. 2009;18(21):4066–80. 10.1093/hmg/ddp355 19640925 PMC2758138

[32] Le NM, Parikh S. Late infantile neuronal ceroid lipofuscinosis and dopamine deficiency. J Child Neurol. 2012;27(2):234–7. 10.1177/0883073811419261 . 21940688

[33] Weimer JM, Benedict JW, Elshatory YM, Short DW, Ramirez-Montealegre D, Ryan DA, et al Alterations in striatal dopamine catabolism precede loss of substantia nigra neurons in a mouse model of juvenile neuronal ceroid lipofuscinosis. Brain research. 2007;1162:98–112. 10.1016/j.brainres.2007.05.018 . 17617387 PMC4790084

[34] Aberg L, Liewendahl K, Nikkinen P, Autti T, Rinne JO, Santavuori P. Decreased striatal dopamine transporter density in JNCL patients with parkinsonian symptoms. Neurology. 2000;54(5):1069–74. . 10720276 10.1212/wnl.54.5.1069

[35] Nijssen PC, Brusse E, Leyten AC, Martin JJ, Teepen JL, Roos RA. Autosomal dominant adult neuronal ceroid lipofuscinosis: parkinsonism due to both striatal and nigral dysfunction. Movement disorders: official journal of the Movement Disorder Society. 2002;17(3):482–7. 10.1002/mds.10104 . 12112194

[36] Kirveskari E, Partinen M, Santavuori P. Sleep and its disturbance in a variant form of late infantile neuronal ceroid lipofuscinosis (CLN5). J Child Neurol. 2001;16(10):707–13. . 11669342 10.1177/088307380101601001

[37] Boustany RM. Lysosomal storage diseases—the horizon expands. Nature reviews Neurology. 2013;9(10):583–98. 10.1038/nrneurol.2013.163 . 23938739

[38] Linterman KS, Palmer DN, Kay GW, Barry LA, Mitchell NL, McFarlane RG, et al Lentiviral-mediated gene transfer to the sheep brain: implications for gene therapy in Batten disease. Human gene therapy. 2011;22(8):1011–20. 10.1089/hum.2011.026 21595499 PMC3159522

[39] Platt FM. Sphingolipid lysosomal storage disorders. Nature. 2014;510(7503):68–75. 10.1038/nature13476 . 24899306

[40] Wong AM, Rahim AA, Waddington SN, Cooper JD. Current therapies for the soluble lysosomal forms of neuronal ceroid lipofuscinosis. Biochemical Society transactions. 2010;38(6):1484–8. 10.1042/BST0381484 . 21118112

[41] Karageorgos L, Lancaster MJ, Nimmo JS, Hopwood JJ. Gaucher disease in sheep. Journal of inherited metabolic disease. 2011;34(1):209–15. 10.1007/s10545-010-9230-3 . 20978939

[42] Suarez-Vega A, Gutierrez-Gil B, Cuchillo-Ibanez I, Saez-Valero J, Perez V, Garcia-Gamez E, et al Identification of a 31-bp deletion in the RELN gene causing lissencephaly with cerebellar hypoplasia in sheep. PloS one. 2013;8(11):e81072 10.1371/journal.pone.0081072 24260534 PMC3834269

[43] Kessell AE, Finnie JW, Manavis J, Cheetham GD, Blumbergs PC. A Rosenthal fiber encephalomyelopathy resembling Alexander's disease in 3 sheep. Veterinary pathology. 2012;49(2):248–54. 10.1177/0300985810395783 . 21233330

[44] Torres PA, Zeng BJ, Porter BF, Alroy J, Horak F, Horak J, et al Tay-Sachs disease in Jacob sheep. Molecular genetics and metabolism. 2010;101(4):357–63. 10.1016/j.ymgme.2010.08.006 . 20817517

[45] Jacobsen JC, Bawden CS, Rudiger SR, McLaughlan CJ, Reid SJ, Waldvogel HJ, et al An ovine transgenic Huntington's disease model. Hum Mol Genet. 2010;19(10):1873–82. 10.1093/hmg/ddq063 20154343 PMC2860888

