# Supplementary figures and images for: Rapid and Progressive Regional Brain Atrophy in CLN6 Batten Disease Affected Sheep Measured with Longitudinal Magnetic Resonance Imaging
Source: PLoS One. 2015 Jul 10;10(7):e0132331. doi: 10.1371/journal.pone.0132331 (PMC4498759; doi:10.1371/journal.pone.0132331)

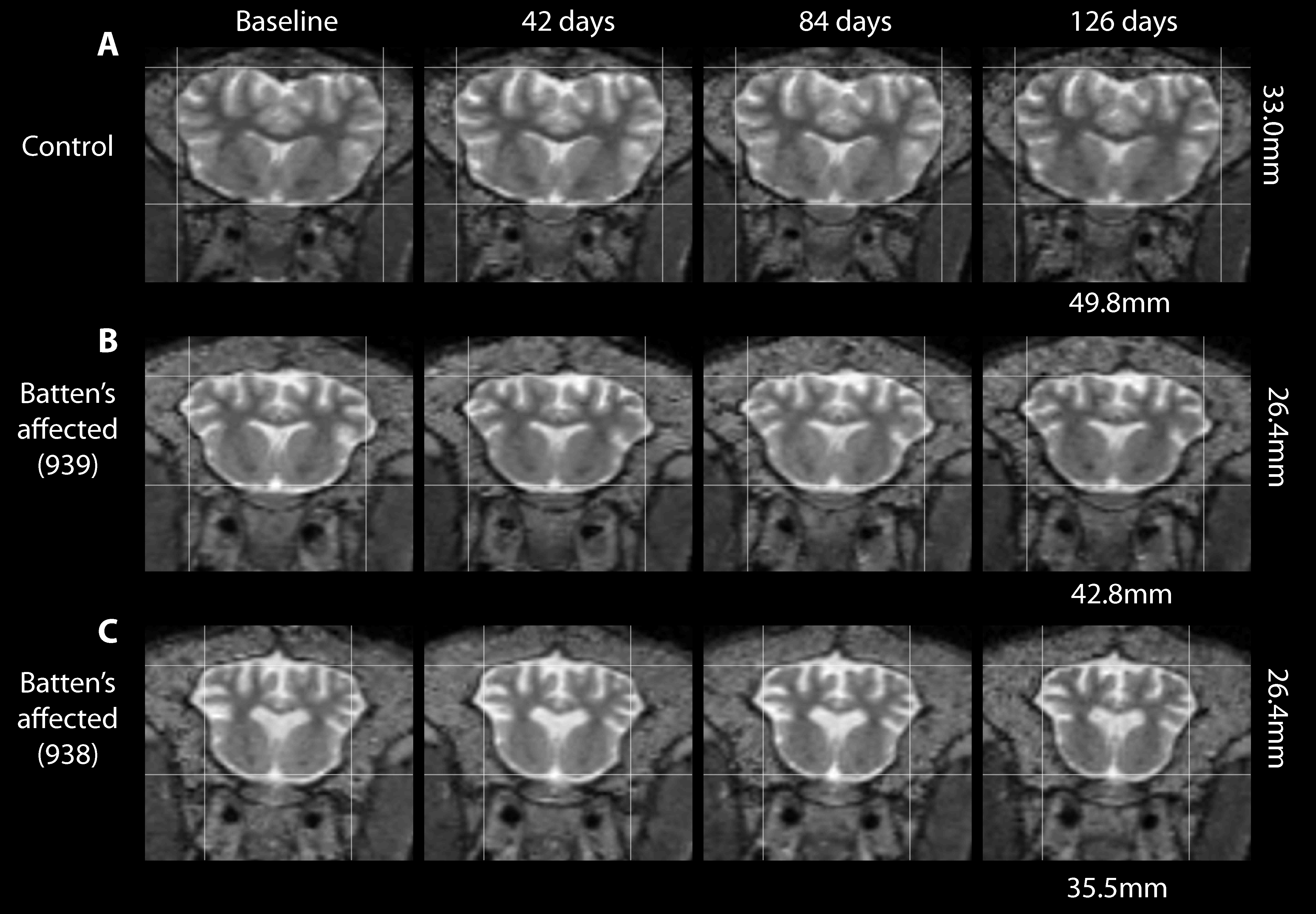

Supplement: S1 Fig — MRI sections showing longitudinal progression in a control sheep (A) and the least- (B) and worst-affected (C) Batten disease affected sheep. To aid comparison in the figure, gridlines were drawn to frame the grey matter surface of each of brain at the last time point, and these lines are reproduced on the earlier scans for comparison. Gridlines on each set for scans are a box enclosing the grey matter surface on the final scan reproduced at each of the earlier time points. Scale bar is 1cm. (TIF) [file pone.0132331.s001.tif]

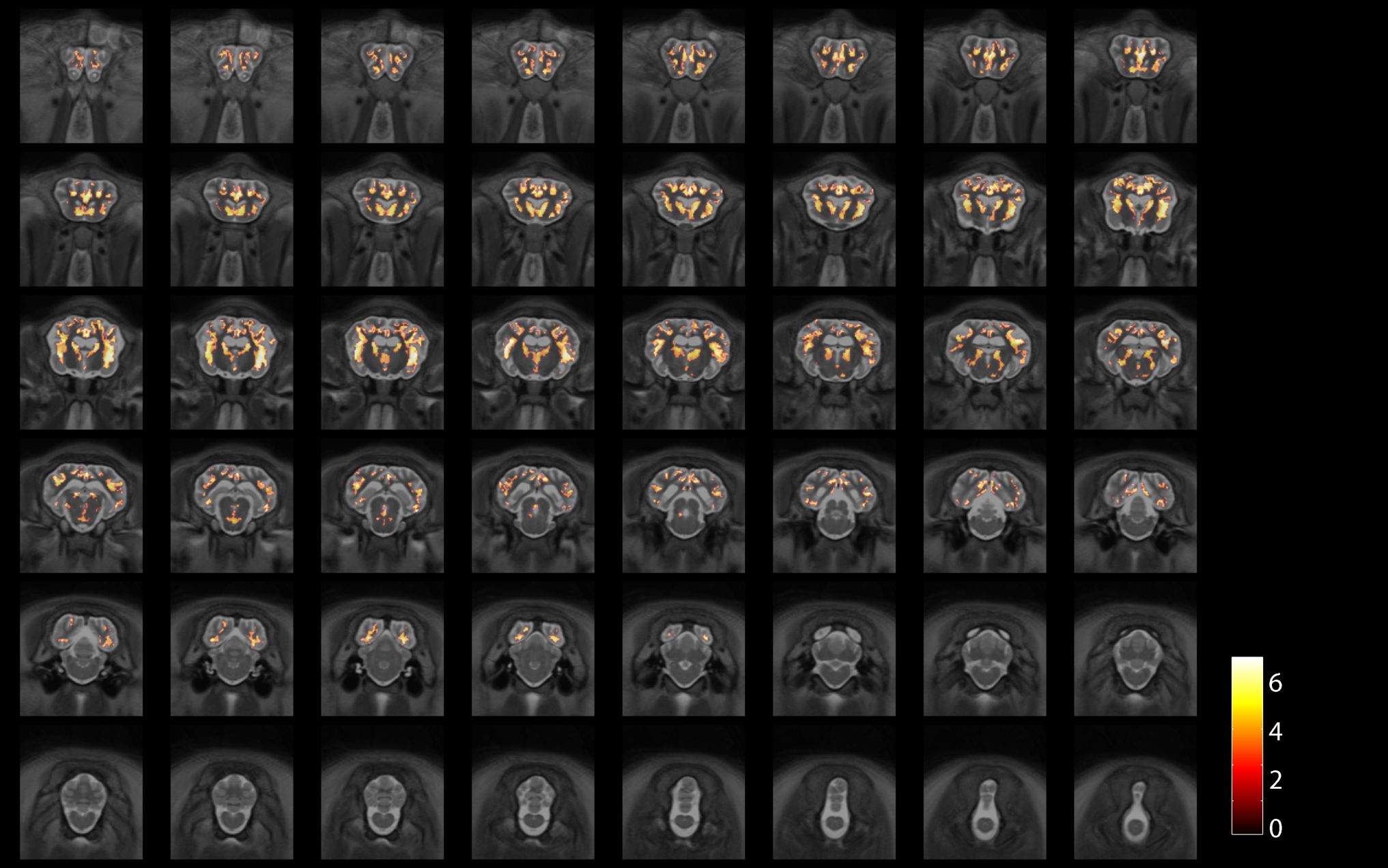

Supplement: S2 Fig — Colour bar indicates Student’s t-score with seven degrees of freedom. All changes shown are significant at p < 0.05 adjusted for multiple comparisons by controlling the false-discovery rate. (TIF) [file pone.0132331.s002.tif]
