# Supplementary material for: Rapid and Progressive Regional Brain Atrophy in CLN6 Batten Disease Affected Sheep Measured with Longitudinal Magnetic Resonance Imaging
Source: PLoS One. 2015 Jul 10;10(7):e0132331. doi: 10.1371/journal.pone.0132331 (PMC4498759; doi:10.1371/journal.pone.0132331)
Supplement: S1 Table — (DOCX) [file pone.0132331.s003.docx]

|  |  |  |  | scan week | | | |  |
| --- | --- | --- | --- | --- | --- | --- | --- | --- |
|  |  |  | scan number | 1 | 2 | 3 | 4 | |
| ID | genotype | sex | Age at first scan  (months) |  |  |  |  | |
| 935 | Het | wether | 18.5 | 1 | 6 | 11 | 17 | |
| 936 | Het | wether | 18.5 | 1 | 6 | 11 | 17 | |
| 942 | Het | wether | 17 | 1 | 8 | 13 | 17 | |
| 944 | Het | wether | 17 | 1 | 9 | 14 | 20 | |
| 937 | Hom | ewe | 18 | 1 | 6 | 12 | 15 | |
| 938 | Hom | wether | 18 | 1 | 7 | 12 | 18 | |
| 939 | Hom | wether | 18 | 1 | 11 | 14 | 20 | |
| 943 | Hom | ewe | 16.5 | 1 | 7 | 12 | 18 | |
| 945 | Hom | ewe | 17 | 1* |  |  |  | |
| 947 | Hom | ewe | 18 | 1 | 6 | 12 | 18 | |

**S1 Table Timeline of MRI scans**

* only one scan performed
Hom = homozygous for *CLN6* mutation (Batten disease affected sheep)
Het = heterozygous for *CLN6* mutation (unaffected control sheep
