# Supplementary material for: Rapid and Progressive Regional Brain Atrophy in CLN6 Batten Disease Affected Sheep Measured with Longitudinal Magnetic Resonance Imaging
Source: PLoS One. 2015 Jul 10;10(7):e0132331. doi: 10.1371/journal.pone.0132331 (PMC4498759; doi:10.1371/journal.pone.0132331)
Supplement: S2 Table — (DOCX) [file pone.0132331.s004.docx]

| ID | Sex | Seizures before scan period | No. of seizures during scan period (spontaneous/ handling-induced/total) | Age at first seizure (months) | Convulsive | age at first scan  (months) | age at last scan (months) |
| --- | --- | --- | --- | --- | --- | --- | --- |
| 938 | wether | >10 | 8/2/10 | ~12 | yes | 18 | 22 |
| 947 | ewe | 0 | 8/1/9 | 20 | yes | 18 | 20.5 |
| 943 | ewe | 0 | 2/1/3 | 18 | no | 18.5 | 22 |
| 937 | ewe | 0 | 0/2/2 | 21 | no | 18.5 | 21.5 |
| 939 | wether | 0 | 0/1/1 | 21 | no | 16.5 | 20.5 |
| *945* | *ewe* | *0* | *0/1/1 (during MRI)* | *21* | *(yes)* | *16.5* | *N/A** |

**Supplementary Table 2.** Seizure onset and severity in homozygous affected Batten sheep

* only one scan performed
