# Supplementary material for: Rapid and Progressive Regional Brain Atrophy in CLN6 Batten Disease Affected Sheep Measured with Longitudinal Magnetic Resonance Imaging
Source: PLoS One. 2015 Jul 10;10(7):e0132331. doi: 10.1371/journal.pone.0132331 (PMC4498759; doi:10.1371/journal.pone.0132331)
Supplement: S3 Table — (DOCX) [file pone.0132331.s005.docx]

**Supplementary Table 3.** Regional volume changes in Batten disease sheep

A. Cerebellum

|  | | Raw volumes / ml | | | | Baseline adjusted volume / ml | | | |
| --- | --- | --- | --- | --- | --- | --- | --- | --- | --- |
| sheep details | | scan number | | | | scan number | | | |
| ID | Genotype | 1 | 2 | 3 | 4 | 1 | 2 | 3 | 4 |
| 935 | Het | 8.83 | 9.13 | 9.18 | 9.21 | 9.25 | 9.55 | 9.60 | 9.63 |
| 936 | Het | 8.67 | 9.33 | 9.49 | 9.48 | 9.25 | 9.91 | 10.08 | 10.06 |
| 942 | Het | 9.75 | 9.53 | 9.50 | 9.66 | 9.25 | 9.03 | 9.00 | 9.16 |
| 944 | Het | 9.76 | 9.72 | 9.11 | 9.88 | 9.25 | 9.22 | 8.61 | 9.38 |
| 937 | Hom | 8.02 | 8.18 | 8.19 | 7.97 | 8.23 | 8.4 | 8.41 | 8.19 |
| 938 | Hom | 8.66 | 8.79 | 8.85 | 8.96 | 8.23 | 8.37 | 8.43 | 8.54 |
| 939 | Hom | 9.06 | 9.20 | 9.26 | 9.31 | 8.23 | 8.38 | 8.44 | 8.49 |
| 943 | Hom | 7.58 | 7.61 | 7.67 | 7.69 | 8.23 | 8.27 | 8.33 | 8.34 |
| 947 | Hom | 7.87 | 7.80 | 7.76 | 7.84 | 8.23 | 8.17 | 8.13 | 8.21 |

B. Lateral ventricles

|  | | | Raw volumes / ml | | | | | Baseline adjusted volume / ml | | | |
| --- | --- | --- | --- | --- | --- | --- | --- | --- | --- | --- | --- |
| sheep details | | scan number | | | | | scan number | | | | |
| ID | Genotype | | 1 | 2 | 3 | 4 | | 1 | 2 | 3 | 4 |
| 935 | Het | | 3.49 | 3.56 | 3.50 | 3.48 | | 3.81 | 3.88 | 3.82 | 3.80 |
| 936 | Het | | 4.40 | 4.39 | 4.43 | 4.31 | | 3.81 | 3.8 | 3.85 | 3.72 |
| 942 | Het | | 4.14 | 4.26 | 4.35 | 4.08 | | 3.81 | 3.93 | 4.02 | 3.75 |
| 944 | Het | | 3.21 | 3.28 | 3.24 | 3.27 | | 3.81 | 3.88 | 3.84 | 3.87 |
| 937 | Hom | | 5.50 | 5.41 | 5.39 | 5.32 | | 4.44 | 4.36 | 4.34 | 4.27 |
| 938 | Hom | | 5.26 | 5.28 | 5.27 | 5.26 | | 4.44 | 4.47 | 4.45 | 4.44 |
| 939 | Hom | | 3.96 | 4.06 | 4.06 | 4.29 | | 4.44 | 4.54 | 4.54 | 4.77 |
| 943 | Hom | | 4.00 | 4.15 | 4.23 | 4.09 | | 4.44 | 4.60 | 4.68 | 4.54 |
| 947 | Hom | | 3.51 | 3.54 | 3.59 | 3.58 | | 4.44 | 4.47 | 4.52 | 4.51 |

C. Caudate

|  | | Raw volumes / ml | | | | Baseline adjusted volume / ml | | | |
| --- | --- | --- | --- | --- | --- | --- | --- | --- | --- |
| sheep details | | scan number | | | | scan number | | | |
| ID | Genotype | 1 | 2 | 3 | 4 | 1 | 2 | 3 | 4 |
| 935 | Het | 0.85 | 0.83 | 0.82 | 0.82 | 0.97 | 0.96 | 0.94 | 0.95 |
| 936 | Het | 1.02 | 1.02 | 1.02 | 1.01 | 0.97 | 0.97 | 0.98 | 0.96 |
| 942 | Het | 1.05 | 1.07 | 1.05 | 1.09 | 0.97 | 1.00 | 0.97 | 1.02 |
| 944 | Het | 0.98 | 0.99 | 0.99 | 1.00 | 0.97 | 0.99 | 0.98 | 1.00 |
| 937 | Hom | 0.64 | 0.62 | 0.58 | 0.57 | 0.7 | 0.68 | 0.64 | 0.63 |
| 938 | Hom | 0.72 | 0.67 | 0.66 | 0.61 | 0.7 | 0.65 | 0.64 | 0.58 |
| 939 | Hom | 0.81 | 0.79 | 0.76 | 0.75 | 0.7 | 0.67 | 0.64 | 0.64 |
| 943 | Hom | 0.65 | 0.64 | 0.60 | 0.59 | 0.7 | 0.68 | 0.64 | 0.64 |
| 947 | Hom | 0.67 | 0.64 | 0.61 | 0.61 | 0.7 | 0.67 | 0.64 | 0.64 |

D. Putamen

|  | | Raw volumes / ml | | | | Baseline adjusted volume / ml | | | |
| --- | --- | --- | --- | --- | --- | --- | --- | --- | --- |
| sheep details | | scan number | | | | scan number | | | |
| ID | Genotype | 1 | 2 | 3 | 4 | 1 | 2 | 3 | 4 |
| 935 | Het | 0.33 | 0.33 | 0.33 | 0.33 | 0.34 | 0.34 | 0.33 | 0.33 |
| 936 | Het | 0.33 | 0.34 | 0.33 | 0.32 | 0.34 | 0.34 | 0.34 | 0.33 |
| 942 | Het | 0.37 | 0.36 | 0.36 | 0.37 | 0.34 | 0.34 | 0.34 | 0.34 |
| 944 | Het | 0.33 | 0.33 | 0.33 | 0.32 | 0.34 | 0.34 | 0.34 | 0.34 |
| 937 | Hom | 0.28 | 0.26 | 0.24 | 0.23 | 0.25 | 0.23 | 0.21 | 0.20 |
| 938 | Hom | 0.27 | 0.27 | 0.27 | 0.25 | 0.25 | 0.25 | 0.24 | 0.23 |
| 939 | Hom | 0.28 | 0.26 | 0.24 | 0.23 | 0.25 | 0.22 | 0.21 | 0.2 |
| 943 | Hom | 0.22 | 0.23 | 0.23 | 0.21 | 0.25 | 0.26 | 0.25 | 0.24 |
| 947 | Hom | 0.19 | 0.23 | 0.21 | 0.20 | 0.25 | 0.29 | 0.27 | 0.26 |

E. Amygdala

|  | | Raw volumes / ml | | | | Baseline adjusted volume / ml | | | |
| --- | --- | --- | --- | --- | --- | --- | --- | --- | --- |
| sheep details | | scan number | | | | scan number | | | |
| ID | Genotype | 1 | 2 | 3 | 4 | 1 | 2 | 3 | 4 |
| 935 | Het | 0.24 | 0.24 | 0.24 | 0.24 | 0.25 | 0.25 | 0.25 | 0.25 |
| 936 | Het | 0.25 | 0.25 | 0.26 | 0.25 | 0.25 | 0.25 | 0.26 | 0.25 |
| 942 | Het | 0.26 | 0.27 | 0.26 | 0.27 | 0.25 | 0.25 | 0.25 | 0.26 |
| 944 | Het | 0.25 | 0.26 | 0.26 | 0.27 | 0.25 | 0.26 | 0.26 | 0.27 |
| 937 | Hom | 0.18 | 0.16 | 0.15 | 0.15 | 0.16 | 0.15 | 0.13 | 0.13 |
| 938 | Hom | 0.18 | 0.17 | 0.17 | 0.16 | 0.16 | 0.16 | 0.15 | 0.14 |
| 939 | Hom | 0.18 | 0.17 | 0.17 | 0.17 | 0.16 | 0.15 | 0.15 | 0.15 |
| 943 | Hom | 0.16 | 0.16 | 0.16 | 0.16 | 0.16 | 0.17 | 0.17 | 0.16 |
| 947 | Hom | 0.12 | 0.12 | 0.11 | 0.11 | 0.16 | 0.16 | 0.16 | 0.16 |

F. Hippocampus

|  | | Raw volumes / ml | | | | Baseline adjusted volume / ml | | | |
| --- | --- | --- | --- | --- | --- | --- | --- | --- | --- |
| sheep details | | scan number | | | | scan number | | | |
| ID | Genotype | 1 | 2 | 3 | 4 | 1 | 2 | 3 | 4 |
| 935 | Het | 1.14 | 1.16 | 1.15 | 1.17 | 1.23 | 1.25 | 1.24 | 1.26 |
| 936 | Het | 1.25 | 1.25 | 1.28 | 1.25 | 1.23 | 1.23 | 1.26 | 1.23 |
| 942 | Het | 1.33 | 1.34 | 1.33 | 1.32 | 1.23 | 1.23 | 1.23 | 1.22 |
| 944 | Het | 1.19 | 1.20 | 1.15 | 1.20 | 1.23 | 1.24 | 1.19 | 1.24 |
| 937 | Hom | 1.14 | 1.12 | 1.11 | 1.10 | 1.01 | 0.99 | 0.99 | 0.97 |
| 938 | Hom | 1.12 | 1.13 | 1.13 | 1.13 | 1.01 | 1.02 | 1.02 | 1.02 |
| 939 | Hom | 1.08 | 1.08 | 1.07 | 1.11 | 1.01 | 1.01 | 1.00 | 1.04 |
| 943 | Hom | 0.89 | 0.88 | 0.88 | 0.91 | 1.01 | 1.00 | 0.99 | 1.03 |
| 947 | Hom | 0.82 | 0.87 | 0.86 | 0.90 | 1.01 | 1.06 | 1.05 | 1.09 |

G. Thalamus

|  | | Raw volumes / ml | | | | Baseline adjusted volume / ml | | | |
| --- | --- | --- | --- | --- | --- | --- | --- | --- | --- |
| sheep details | | scan number | | | | scan number | | | |
| ID | Genotype | 1 | 2 | 3 | 4 | 1 | 2 | 3 | 4 |
| 935 | Het | 2.57 | 2.61 | 2.65 | 2.70 | 2.65 | 2.68 | 2.73 | 2.78 |
| 936 | Het | 2.46 | 2.58 | 2.6 | 2.55 | 2.65 | 2.77 | 2.78 | 2.74 |
| 942 | Het | 2.97 | 2.93 | 2.93 | 2.98 | 2.65 | 2.60 | 2.60 | 2.66 |
| 944 | Het | 2.58 | 2.61 | 2.52 | 2.62 | 2.65 | 2.67 | 2.59 | 2.68 |
| 937 | Hom | 1.90 | 1.88 | 1.78 | 1.70 | 1.90 | 1.87 | 1.78 | 1.70 |
| 938 | Hom | 1.94 | 1.95 | 1.91 | 1.82 | 1.90 | 1.91 | 1.87 | 1.78 |
| 939 | Hom | 2.25 | 2.19 | 2.17 | 2.09 | 1.90 | 1.83 | 1.82 | 1.73 |
| 943 | Hom | 1.81 | 1.82 | 1.74 | 1.73 | 1.90 | 1.91 | 1.83 | 1.82 |
| 947 | Hom | 1.59 | 1.61 | 1.55 | 1.58 | 1.90 | 1.92 | 1.86 | 1.89 |

H. Globus pallidus

|  | | Raw volumes / ml | | | | | | | Baseline adjusted volume / ml | | | | | | |
| --- | --- | --- | --- | --- | --- | --- | --- | --- | --- | --- | --- | --- | --- | --- | --- |
| sheep details | | scan number | | | | | | | scan number | | | | | | |
| ID | Genotype | 1 | | 2 | | 3 | | 4 | 1 | | 2 | | 3 | | 4 |
| 935 | Het | 0.18 | | 0.18 | | 0.18 | | 0.18 | 0.19 | | 0.20 | | 0.20 | | 0.19 |
| 936 | Het | 0.18 | | 0.19 | | 0.19 | | 0.18 | 0.19 | | 0.20 | | 0.20 | | 0.19 |
| 942 | Het | 0.20 | | 0.20 | | 0.20 | | 0.20 | 0.19 | | 0.19 | | 0.19 | | 0.19 |
| 944 | Het | 0.21 | | 0.21 | | 0.20 | | 0.21 | 0.19 | | 0.19 | | 0.18 | | 0.19 |
| 937 | Hom | 0.17 | | 0.16 | | 0.15 | | 0.14 | 0.15 | | 0.14 | | 0.13 | | 0.13 |
| 938 | Hom | 0.15 | | 0.15 | | 0.15 | | 0.15 | 0.15 | | 0.15 | | 0.15 | | 0.15 |
| 939 | Hom | 0.18 | | 0.17 | | 0.17 | | 0.16 | 0.15 | | 0.14 | | 0.14 | | 0.13 |
| 943 | Hom | 0.14 | 0.15 | | 0.15 | | 0.15 | | 0.15 | 0.16 | | 0.16 | | 0.16 | |
| 947 | Hom | 0.11 | 0.13 | | 0.12 | | 0.12 | | 0.15 | 0.17 | | 0.16 | | 0.16 | |
